# Portland Intensive Insulin Therapy During Living Donor Liver Transplantation: Association with Postreperfusion Hyperglycemia and Clinical Outcomes

**DOI:** 10.1038/s41598-018-34655-6

**Published:** 2018-11-02

**Authors:** RyungA Kang, Sangbin Han, Kyo Won Lee, Gaab Soo Kim, Soo Joo Choi, Justin S. Ko, Sang Hyun Lee, Mi Sook Gwak

**Affiliations:** 1 Department of Anesthesiology and Pain Medicine, Samsung Medical Center, Sungkyunkwan University School of Medicine, Seoul, Korea; 2 Department of Surgery, Samsung Medical Center, Sungkyunkwan University School of Medicine, Seoul, Korea

## Abstract

Many liver transplant recipients experience intraoperative hyperglycemia after graft reperfusion. Accordingly, we introduced the Portland intensive insulin therapy (PoIIT) in our practice to better control blood glucose concentration (BGC). We evaluated the effects of PoIIT by comparing with our conventional insulin therapy (CoIT). Of 128 patients who underwent living donor liver transplantation (LDLT) during the phaseout period of CoIT, 89 were treated with the PoIIT and 39 were treated with CoIT. The primary outcome was hyperglycemia (BGC > 180 mg/dL) during the intraoperative postreperfusion phase. The secondary outcomes were postoperative complications such as infection. The incidence of hyperglycemia (22.5% vs. 53.8%, p = 0.001) and prolonged hyperglycemia for >2 hours (7.9% vs. 30.8%, p = 0.002) was significantly lower in PoIIT group than in CoIT group. A mixed linear model further demonstrated that repeatedly measured BGCs were lower in PoIIT group (p < 0.001). The use of PoIIT was significantly associated with decreases in major infections (OR = 0.23 [0.06–0.85], p = 0.028), prolonged mechanical ventilation (OR = 0.29 [0.09–0.89], p = 0.031), and biliary stricture (OR = 0.23 [0.07–0.78], p = 0.018) after adjustments for age, sex, and diabetes mellitus. In conclusion, the PoIIT is effective for maintaining BGC and preventing hyperglycemia during the intraoperative postreperfusion phase of living donor liver transplantation with potential clinical benefits.

## Introduction

Liver transplantation is attended with various metabolic disturbances^1^. Rapid increase in blood glucose concentration (BGC) after graft reperfusion is one of them and results from the systemic influx of glucose from hepatocytes destroyed by ischemia reperfusion injury^2,3^. Intraoperative hyperglycemia during liver transplantation is associated with surgical site infection, overall infectious complications, and mortality^4–6^. The decrease in immunity^7,8^ and increase in ischemia reperfusion injury^9^ resulting from acute hyperglycemic disturbance are thought to be the underlying mechanisms for these adverse outcomes. Moreover, the early postreperfusion period is a critical time during which substantial hepatic insult occurs and graft regeneration is initiated concurrent with metabolic, synthetic, and detoxifying burdens^10–12^. Accordingly, liver transplant anesthesiologists strive to maintain glycemic homeostasis during the intraoperative postreperfusion phase to improve post-transplant clinical courses. However, hyperglycemia is still not rare because the extent of hepatocyte ischemia reperfusion injury is profound and insulin sensitivity is impaired due to underlying chronic liver disease, surgical stress, vasoactive drugs, and steroid immunosuppression. The optimal postreperfusion glycemic strategies to improve liver transplant outcomes remain unresolved^6^.

As a well-established form of intensive insulin therapy (IIT), the Portland IIT (PoIIT) protocol was designed for patients treated in surgical cardiac intensive care units (ICU) and validated as a reliable glycemic strategy with clinical benefits^13,14^. The success of the PoIIT is attributable to a dynamic algorithm modifying therapeutic insulin doses by accounting for patient insulin sensitivity that is evaluated according to the rate of BGC change at a set insulin dose. That is, calculated insulin dose differs according to the rate of BGC change even if the measured BGCs are the same^15^. Thus, we had deduced that the PoIIT effectively controls rapid intraoperative hyperglycemic changes in liver transplant recipients and introduced the PoIIT to our practice in January 2015. Among various algorithms with different target BGC ranges, we chose the PoIIT with the lowest target range (80–120 mg/dL) to rapidly control hyperglycemia and minimize the duration of intraoperative hyperglycemia^16^. We also considered that the risk of hypoglycemia is low due to frequent intraoperative BGC checkups^17^. Herein, we evaluated the effects of the PoIIT by comparing results with those of our conventional insulin therapy (CoIT) in recipients who underwent liver transplantation during the phaseout period of CoIT.

## Materials and Methods

### Subjects and data collection

We reviewed the medical records of 128 patients who underwent their first adult-to-adult living donor liver transplantations at our institution during the phaseout period of CoIT (implantation period of the PoIIT) between January 2015 and July 2017. Of these, 39 recipients were treated with CoIT and 89 recipients were treated with the PoIIT. During the phaseout period, the PoIIT was gradually established as a standard protocol by ensuring patient safety and compliance with attending anesthetists (Supplementary Table S1). All data were collected from computerized medical records or a liver transplant database (prospectively collected) and were anonymized and de-identified prior to analysis. The Institutional Review Board of Samsung Medical Center approved this retrospective study (SMC 2016-11-016) and waived the requirement for written informed consent. All procedures in this study were performed in accordance with the relevant guidelines and regulations. No grafts were procured from prisoners. All surgeries were performed at our hospital by surgeons in the Department of surgery, Samsung Medical Center.

### Perioperative glycemic management

Recipients fasted starting the evening before surgery and 5% dextrose solution was infused at a rate of 80 mL/h during the fasting period. Preoperative oral carbohydrate supplements were not provided. During surgery, arterial BGCs were measured in combination with other arterial blood-derived parameters using a blood gas/chemistry analysis device (RAPIDLAB1265, Siemens Healthcare Diagnostics Inc., Berlin, Germany) on an hourly basis, as well as at the following additional time points: start of the anhepatic phase, immediately before graft reperfusion, and 5 and 30 minutes after graft reperfusion, as described previously^11^. The blood gas/chemistry analysis device was near each patient and clinicians were able to reach the device within 1 minute after arterial blood sampling. Insulin therapy was not indicated for controlling BGC before graft reperfusion because BGC generally decreases during the anhepatic phase due to the lack of hepatic glucose production^18^. Instead, hyperkalemia was corrected using the conventional large-bolus insulin method (10 units of regular insulin) if the blood potassium concentration was >4.5 mEq/L^19^. After graft reperfusion, the PoIIT or CoIT was initiated at the discretion of anesthetists until ICU arrival. For CoIT, the continuous insulin infusion dose was determined solely by the current BGC: insulin (Humulin R, Eli Lilly, Indianapolis, IN, USA) was infused at 5 U/h if BGC > 150 mg/dL or at 10 U/h if BGC > 200 mg/dL. For PoIIT, both bolus injection and continuous infusion doses were determined by accounting for the degree of BGC change between the current and previous checkups and previous insulin dose in addition to the current BGC (Supplementary Fig. S1)^16^. After ICU arrival, glycemic management was performed by liver transplant surgeons based on a standardized protocol that is independent from intraoperative glycemic protocols. BGCs were measured at ICU arrival and every 8 hours thereafter using a blood gas/chemistry analysis device (RAPIDLAB1265). Insulin dose was increased by 2 U/h if BGC was 180–240 mg/dL and by 3 U/h if BGC was 240–300 mg/dL; in contrast, the insulin dose was decreased by 1 U/h if BGC was 100–140 mg/dL. Insulin therapy was stopped if BGC was <100 mg/dL.

### Anesthetic management

Anesthesia was performed based on a standardized institutional protocol as described previously^11^. In short, mechanical ventilation was delivered at a tidal volume of 8 mL per ideal body weight (kg) using a mixture of medical air and oxygen with positive end-expiratory pressure set at 6 mmHg. The respiratory rate was adjusted as needed to maintain normocapnea. Vasoactive drugs were used to maintain mean arterial pressure >70 mmHg. Metabolic acidosis was corrected with sodium bicarbonate when the base deficit was >10 mEq/L. Body core temperature was maintained using a whole body-sized warm blanket, airway humidifiers, and fluid warming devices. Transfusion of allogeneic blood was strictly controlled based on a restrictive and prophylactic policy, with each blood component transfused separately according to its respective indication. Blood salvage was routinely used for intraoperative autotransfusion irrespective of the presence of a hepatic tumor^20^.

### Perioperative surgical procedures

Acceptance criteria for liver donation were age ≤65 years, body mass index <35 kg/m^2^, macrosteatosis ≤30%, and residual liver volume ≥30%. Individuals with any type of hepatitis or fibrosis were excluded from donation. All grafts consisted of segments 5–8 excluding the middle hepatic vein trunk. Graft implantation was performed using the piggyback technique. After the portal vein anastomosis was completed, the graft was reperfused by consecutively unclamping the hepatic vein and portal vein. The hepatic artery was subsequently anastomosed, followed by biliary anastomosis. Immunosuppression was performed based on a quadruple regimen consisting of methylprednisolone, basiliximab, mycophenolate mofetil, and tacrolimus as described previously^21^. In particularly, recipients received methylprednisolone 500 mg intravenously before graft reperfusion for the induction of immunosuppression and 500 mg daily until postoperative day 2, followed by a tapered dose of 60 mg per day for 5 days, and then 8 mg twice per day for 1 month.

### Variables and statistical analysis

The primary outcome was hyperglycemia during the intraoperative postreperfusion phase. Hyperglycemia was defined when a BGC was >180 mg/dL based on previous research^22^. Secondary outcomes were blood potassium, the extent of hepatocyte injury, and postoperative complications. Hepatocyte injury was determined by monitoring aspartate transaminase (AST) and alanine transaminase (ALT) during the first week after surgery^23^. Postoperative complications were graded according to the modified Clavien-Dindo classification^24,25^. Surgical site infections included superficial/deep incisional infections or organ/space infections according to the Centers for Disease Control definitions for surgical infection^26^. Major infections included septicemia, peritonitis, pneumonia, and tissue-invasive cytomegalovirus disease. During the postoperative period, the first BGC or potassium concentration measured each day was used for analysis. Continuous variables are expressed as mean ± standard deviation or median (25th percentile–75th percentile) and were analyzed by t-test or the Mann-Whitney test. Repeatedly measured continuous variables were analyzed by a mixed linear model with the Bonferroni correction. Categorical variables are expressed as frequency (%) and were analyzed by chi-square test, Fisher’s exact test, or binary logistic regression, as appropriate. In terms of postoperative complications, potential compounding effect from age, sex, and diabetes mellitus was adjusted during multivariable analysis and the risk of type I error or the false discovery rate was controlled by using the Benjamini-Hochberg procedure^27^. All reported p values were 2-sided, and p < 0.05 was considered statistically significant. Analyses were performed using SPSS version 23.0 (IBM, Armonk, NY, USA) or SAS version 9.2 (SAS institute, Cary, NC, USA).

## Results

### Clinical characteristics

As shown in Tables 1 and 2, there were no significant differences between CoIT group and PoIIT group regarding graft quantity/quality, recipient baseline clinical characteristics, or intraoperative hemodynamic variables (p > 0.05). Preoperative fasting BGC was 118 ± 50 mg/dL in CoIT group and 107 ± 32 mg/dL in PoIIT group (p = 0.215). Mean duration of the intraoperative postreperfusion phase was about 4 hours for each group and the amount of insulin infused during the postreperfusion phase was significantly greater in the PoIIT group than in the CoIT group (24 ± 16 U vs. 18 ± 12 U, p = 0.006). No recipients experienced hypoglycemia of BGC < 60 mg/dL^15,28^: only one PoIIT recipient reached <70 mg/dL (65 mg/dL).

**Table 1:** Graft factors, recipient baseline characteristics, and operative variables.

	Conventional (n = 39)	Portland (n = 89)	P
**Graft Factors**			
Age (years)	32 (25–39)	29 (22–38)	0.848
Male sex	24 (61.5)	49 (55.1)	0.563
Macrosteatosis	14 (35.9)	31 (34.8)	>0.99
Graft-to-recipient weight ratio (%)	1.1 ± 0.3	1.0 ± 0.3	0.072
Cold ischemia time (minutes)	94 ± 20	92 ± 23	0.744
Warm ischemia time (minutes)	39 ± 14	43 ± 18	0.184
**Recipient Factors**			
Age (years)	56 (51–62)	56 (52–60)	0.630
Male sex	28 (71.8)	65 (73.0)	>0.99
Body mass index (kg/m^2^)	24 ± 4	25 ± 4	0.541
MELD score	17 ± 10	14 ± 8	0.102
Pre-transplant diabetes	11 (28.9)	18 (20.2)	0.362
Oral hypoglycemic agents	8 (72.7)	15 (83.3)	
Insulin	3 (27.3)	3 (15.8)	
Hypertension	2 (5.1)	11 (12.4)	0.341
Primary etiology			0.242
Hepatitis B virus	26 (66.7)	59 (66.3)	
Hepatitis C virus	0	5 (5.6)	
Alcoholic	3 (7.7)	11 (12.4)	
Others	10 (25.6)	14 (15.7)	
Preoperative laboratory findings			
Glucose (mg/dL)	118 ± 50	107 ± 32	0.215
Potassium (mEq/L)	4.0 ± 0.4	4.1 ± 0.4	0.117
Albumin (g/dL)	3.4 ± 0.6	3.4 ± 0.6	0.800
Creatinine (mg/dL)	0.9 ± 0.4	0.9 ± 0.3	0.272
Sodium (mmol/L)	138 ± 7	139 ± 5	0.317
High sensitivity CRP (mg/dL)	0.5 ± 0.5	0.7 ± 1.5	0.390
Platelet count (×10^9^/L)	85 ± 48	73 ± 44	0.205
Neutrophil-to-lymphocyte ratio	2.9 ± 2.5	3.2 ± 4.1	0.730
Lactate (mmol/L)	2.2 ± 2.1	1.4 ± 0.6	0.082
Aspartate transaminase (U/L)	80 ± 140	53 ± 41	0.099
Alanine transaminase (U/L)	91 ± 280	45 ± 58	0.145
Prothrombin time (INR)	1.8 ± 1.4	1.5 ± 0.6	0.100
Total bilirubin (mg/dL)	6.5 ± 11.5	5.1 ± 4.1	0.483
**Intraoperative factors**			
Vasoactive drug use			
Maximum dopamine dose (µg/kg/min)	0 (0–5)	3 (0–5)	0.222
Maximum norepinephrine dose (µg/kg/min)	0.15 (0.06–0.25)	0.20 (0.10–0.30)	0.682
Maximum vasopressin dose (U/min)	0 (0-0)	0 (0-0)	0.155
Allogeneic transfusion			
Red blood cells (units)	0 (0–2)	0 (0–2)	0.885
Fresh frozen plasma (units)	0 (0–2)	0 (0–2)	0.458
Apheresis platelets (units)	0 (0–0)	0 (0-0)	0.240
Cryoprecipitate (units)	0 (0–4)	0 (0–6)	0.398
Crystalloid infusion (mL)	5780 ± 3373	5735 ± 3175	0.942
Colloid infusion (mL)	1722 ± 695	1871 ± 795	0.313
Anesthesia time (minutes)	610 ± 106	617 ± 94	0.721
Operation time (minutes)	514 ± 105	523 ± 97	0.649
Reperfusion phase time (minutes)	254 ± 71	246 ± 68	0.533
Insulin use before reperfusion	15 (38.5)	26 (29.2)	0.311

Data are presented as mean ± standard deviation or median (25th percentile–75th percentile) for continuous variables and number (%) as appropriate for categorical variables. CRP, C-reactive protein; MELD, model for end-stage liver disease.

**Table 2:** Intraoperative biochemical and hemodynamic variables before and after graft reperfusion.

	Before graft reperfusion*			After graft reperfusion^†^		
	Conventional (n = 39)	Portland (n = 89)	P	Conventional (n = 39)	Portland (n = 89)	P
**Arterial blood gas/chemistry analysis**						
PaO_2_-to-FiO_2_ ratio (mmHg)	557 ± 156	511 ± 150	0.119	430 ± 86	448 ± 107	0.383
pH	7.30 ± 0.06	7.29 ± 0.07	0.426	7.31 ± 0.06	7.30 ± 0.06	0.216
Base excess (mEq/L)	−6.2 ± 3.2	−7.2 ± 3.4	0.153	−6.6 ± 3.2	−7.2 ± 3.3	0.378
Chloride (mEq/L)	108 ± 19	109 ± 4	0.735	109 ± 19	110 ± 4	0.678
**Central laboratory findings**						
Hematocrit (mg/dL)	27 ± 4	27 ± 5	0.546	27 ± 3	27 ± 4	0.944
Platelet count (×10^9^/L)	69 ± 48	62 ± 40	0.546	75 ± 52	70 ± 46	0.235
Prothrombin time (INR)	2.9 ± 1.5	3.1 ± 1.4	0.510	2.9 ± 0.8	3.2 ± 1.3	0.615
Fibrinogen (mg/dL)	120 ± 50	109 ± 47	0.238	110 ± 42	100 ± 37	0.217
**Hemodynamics**						
Mean femoral arterial pressure (mmHg)	67 ± 25	73 ± 21	0.171	71 ± 14	74 ± 13	0.296
Jugular venous pressure (mmHg)	6 ± 3	9 ± 12	0.165	2 ± 4	2 ± 2	0.781
Mean pulmonary artery pressure (mmHg)	16 ± 5	15 ± 5	0.654	16 ± 3	14 ± 4	0.114
Pulmonary capillary wedge pressure (mmHg)	11 ± 3	10 ± 4	0.489	10 ± 3	9 ± 3	0.116
Systemic vascular resistance (dyn·s/cm^5^)	789 ± 241	733 ± 291	0.305	643 ± 221	618 ± 158	0.462
Heart rate (/min)	93 ± 21	94 ± 19	0.873	94 ± 14	97 ± 13	0.423
Cardiac output (L/min)	7.6 ± 1.7	7.9 ± 2.4	0.327	8.2 ± 1.7	8.9 ± 2.2	0.121
Ejection fraction (%)	39 ± 9	42 ± 9	0.250	43 ± 7	44 ± 7	0.613
Mixed venous oxygen saturation (%)	89 ± 6	92 ± 5	0.092	89 ± 6	89 ± 5	0.942

Data are presented as mean ± standard deviation or median (25th percentile–75th percentile). FiO_2_, fraction of inspired oxygen; PaO_2_, arterial partial oxygen pressure. *Immediately before graft reperfusion. ^†^1, 2, 3, and 4 hours after graft reperfusion (mean value).

### Intraoperative BGCs after graft reperfusion

**Figure 1:**
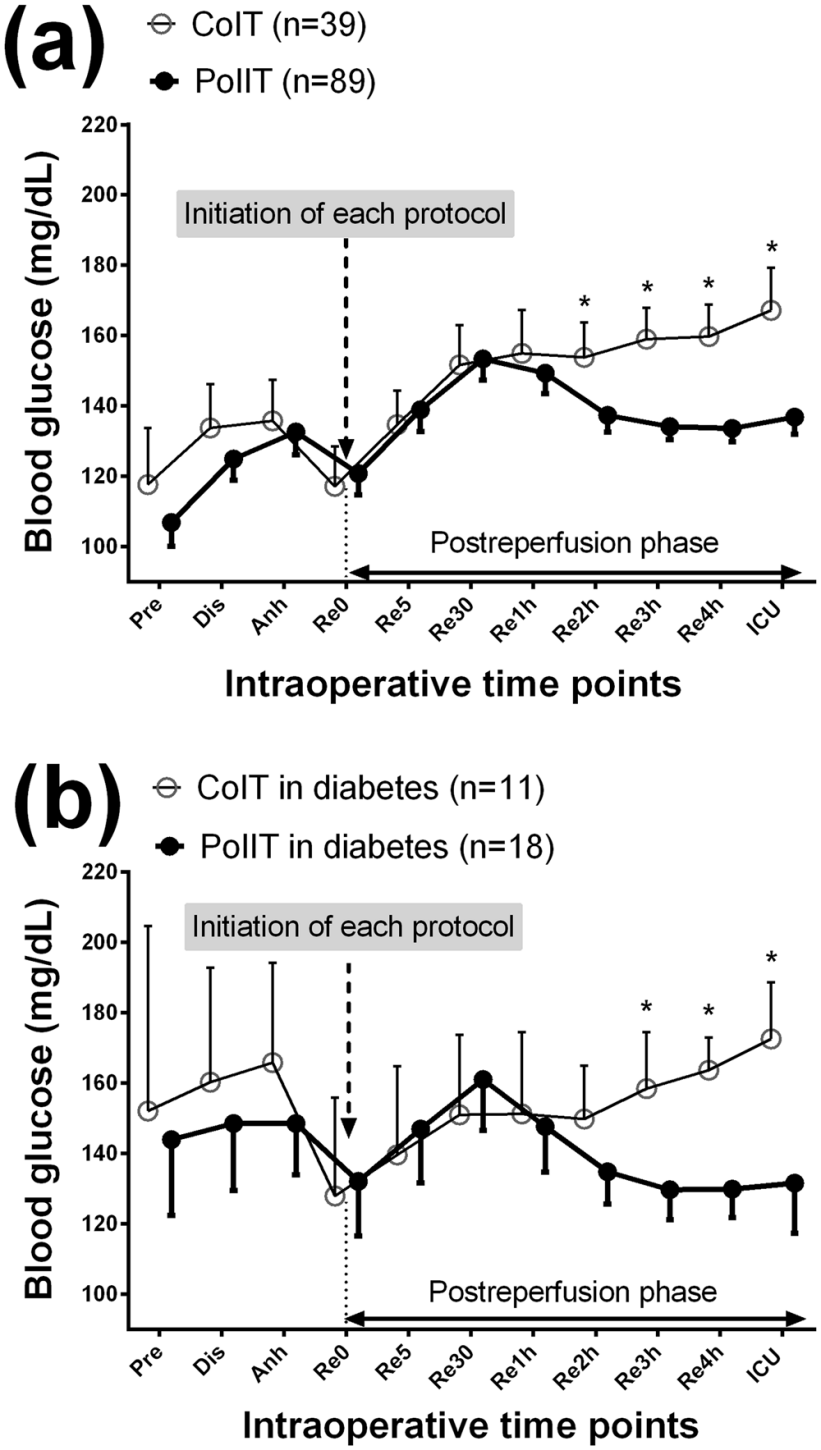
Blood glucose concentrations in (**a**) all recipients and (**b**) diabetic recipients at the following time points; Pre, one day before surgery; Dis, the start of the dissection phase; Anh, the start of the anhepatic phase; Re0, immediately before graft reperfusion; Re5/30, 5/30 minutes after graft reperfusion; Re1h/2 h/3 h/4 h, 1/2/3/4 hours after graft reperfusion; ICU, ICU arrival. *p < 0.05 after Bonferroni correction in mixed linear model analysis.

**Figure 2:**
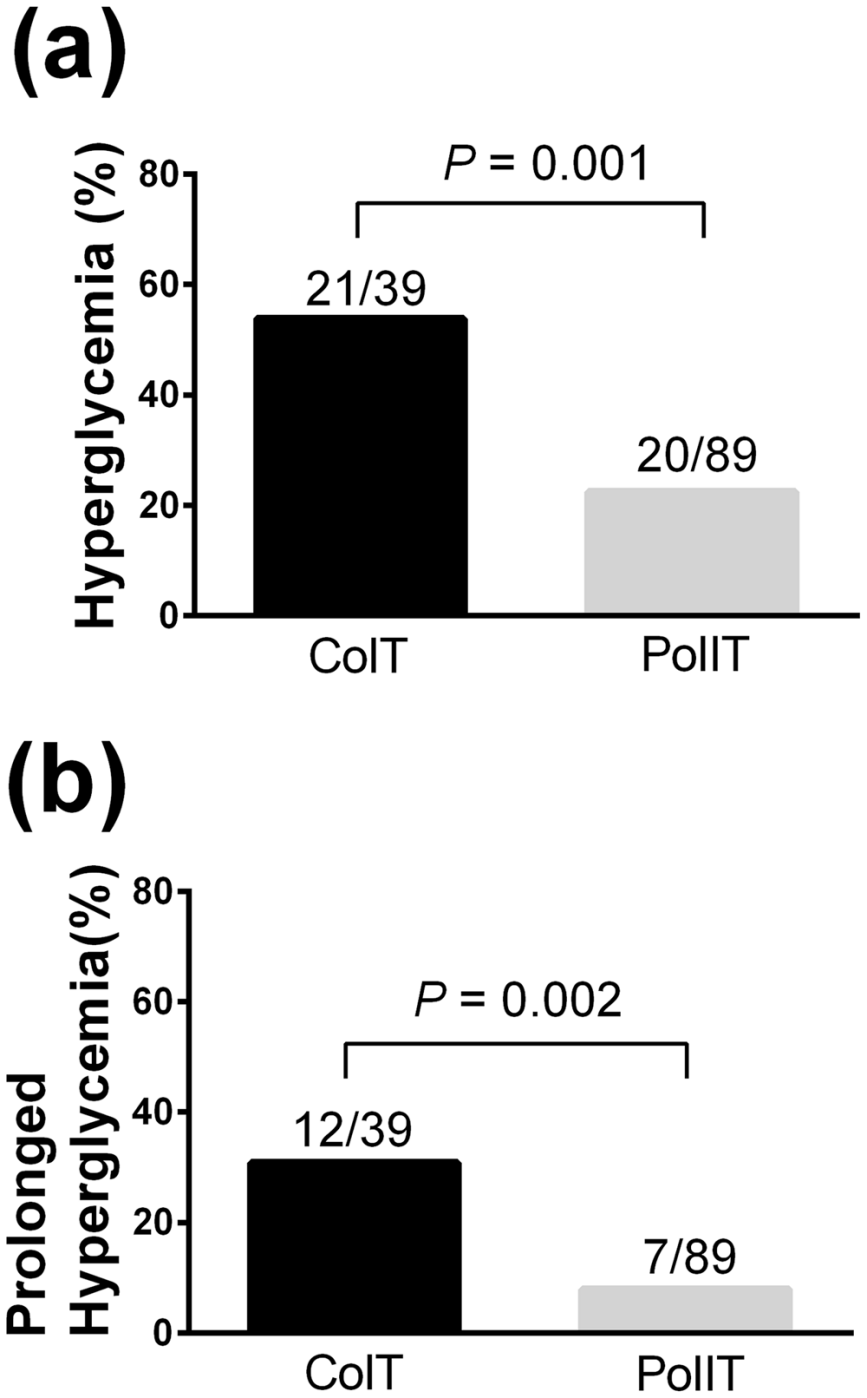
The incidences of (**a**) hyperglycemia and (**b**) prolonged hyperglycemia occurring for >2 hours. The number above the bar indicates the event frequency per group.

BGC at the moment of graft reperfusion was comparable between the two groups: 117 ± 35 mg/dL in CoIT group and 120 ± 28 mg/dL in PoIIT group (p = 0.631). As shown in Fig. 1a, BGCs continued to increase after graft reperfusion in CoIT group, whereas BGCs gradually decreased in PoIIT group. BGCs at 1, 2, 3, and 4 hours after graft reperfusion, and at the moment of ICU arrival, were 155 ± 38, 154 ± 31, 159 ± 28, 160 ± 28, and 167 ± 37 mg/dL, respectively, in CoIT group and 149 ± 27 (p > 0.99), 137 ± 22 (p = 0.015), 134 ± 17 (p < 0.001), 134 ± 17 (p < 0.001), and 137 ± 23 mg/dL (p < 0.001), respectively, in PoIIT group. Repeatedly measured BGCs were significantly lower in PoIIT group than in CoIT group (p < 0.001). As shown in Fig. 2, the incidence of hyperglycemia was significantly lower in PoIIT group than in CoIT group (22.5% vs. 53.8%, p = 0.001) as well as prolonged hyperglycemia occurring during >2 hours (7.9% vs. 30.8%, p = 0.002). At 3 hours after graft reperfusion, no patients in PoIIT group and 9 recipients (23.1%) in CoIT group had hyperglycemia (p < 0.001). Within the subgroup of recipients with diabetes (18 with the PoIIT and 11 with CoIT), BGCs continued to increase after graft reperfusion in CoIT group, whereas BGCs gradually decreased in PoIIT group, as shown in the whole cohort (Fig. 1b). Repeatedly measured BGCs were significantly lower in PoIIT group (p < 0.001). The incidence of hyperglycemia (33.3% vs. 63.6%, p = 0.143) and prolonged hyperglycemia (11.1% vs. 36.4%, p = 0.164) were insignificantly lower in PoIIT group, most likely due to insufficient sample size^29^.

### Intraoperative blood potassium concentrations after graft reperfusion

**Figure 3:**
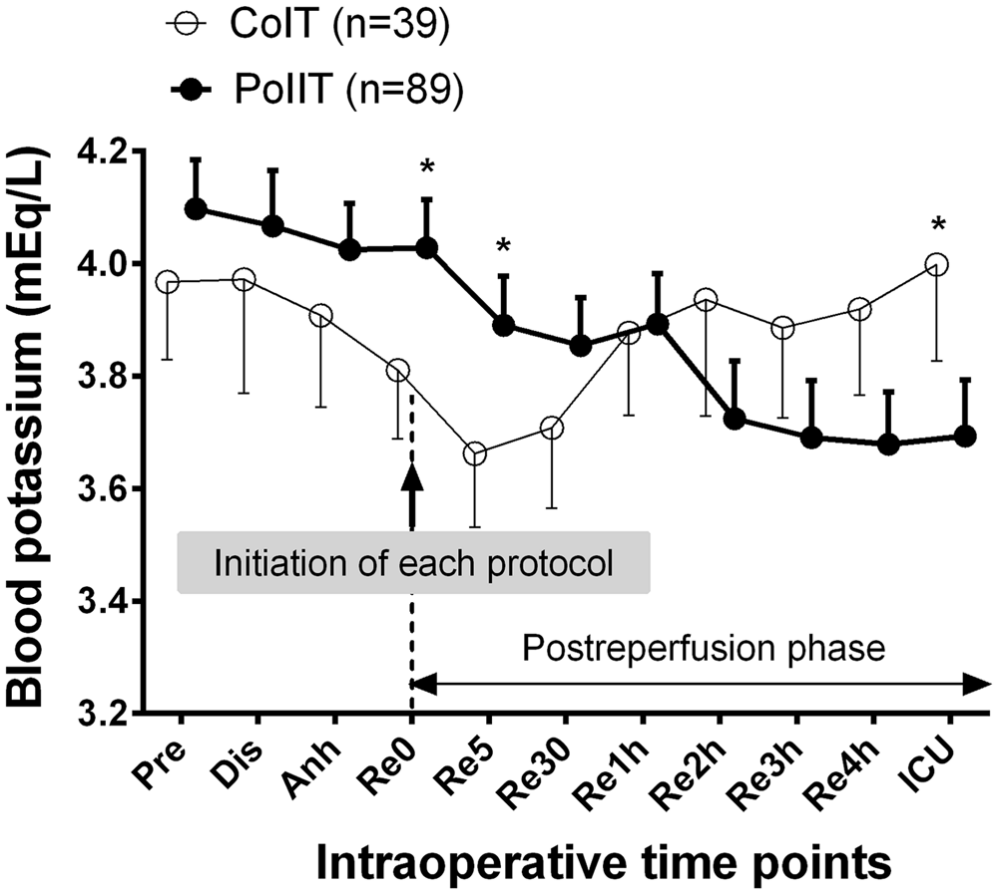
Blood potassium concentrations at the following time points; Pre, one day before surgery; Dis, the start of the dissection phase; Anh, the start of the anhepatic phase; Re0, immediately before graft reperfusion; Re5/30, 5/30 minutes after graft reperfusion; Re1h/2 h/3 h/4 h, 1/2/3/4 hours after graft reperfusion; ICU, ICU arrival. *p < 0.05 after Bonferroni correction in mixed linear model analysis.

Blood potassium concentrations gradually decreased in PoIIT group after graft reperfusion, whereas it continued to increase in CoIT group (Fig. 3). Repeatedly measured potassium concentrations were significantly lower in PoIIT group than in CoIT group (p < 0.001). One CoIT recipient (2.6%) and one PoIIT recipient (1.1%) experienced hyperkalemia (>5.5 mEq/L) during the intraoperative postreperfusion phase (p = 0.518), and were managed with an additional bolus insulin injection. Four CoIT recipients (10.3%) and 6 PoIIT recipients (6.7%) experienced hypokalemia (<3.0 mEq/L, p = 0.492), and were managed with intravenous potassium replacement (5 mEq/h) without any detected complications. No patients reached <2.5 mEq/L.

### Postoperative blood glucose and potassium concentrations

There were no significant differences between the two groups regarding blood glucose and potassium concentrations after the cessation of intraoperative glycemic protocols and the start of the ICU glycemic protocol (Supplementary Fig. S2), indicating that a single ICU glycemic protocol was performed similarly for the two groups.

### Postoperative complications

**Figure 4:**
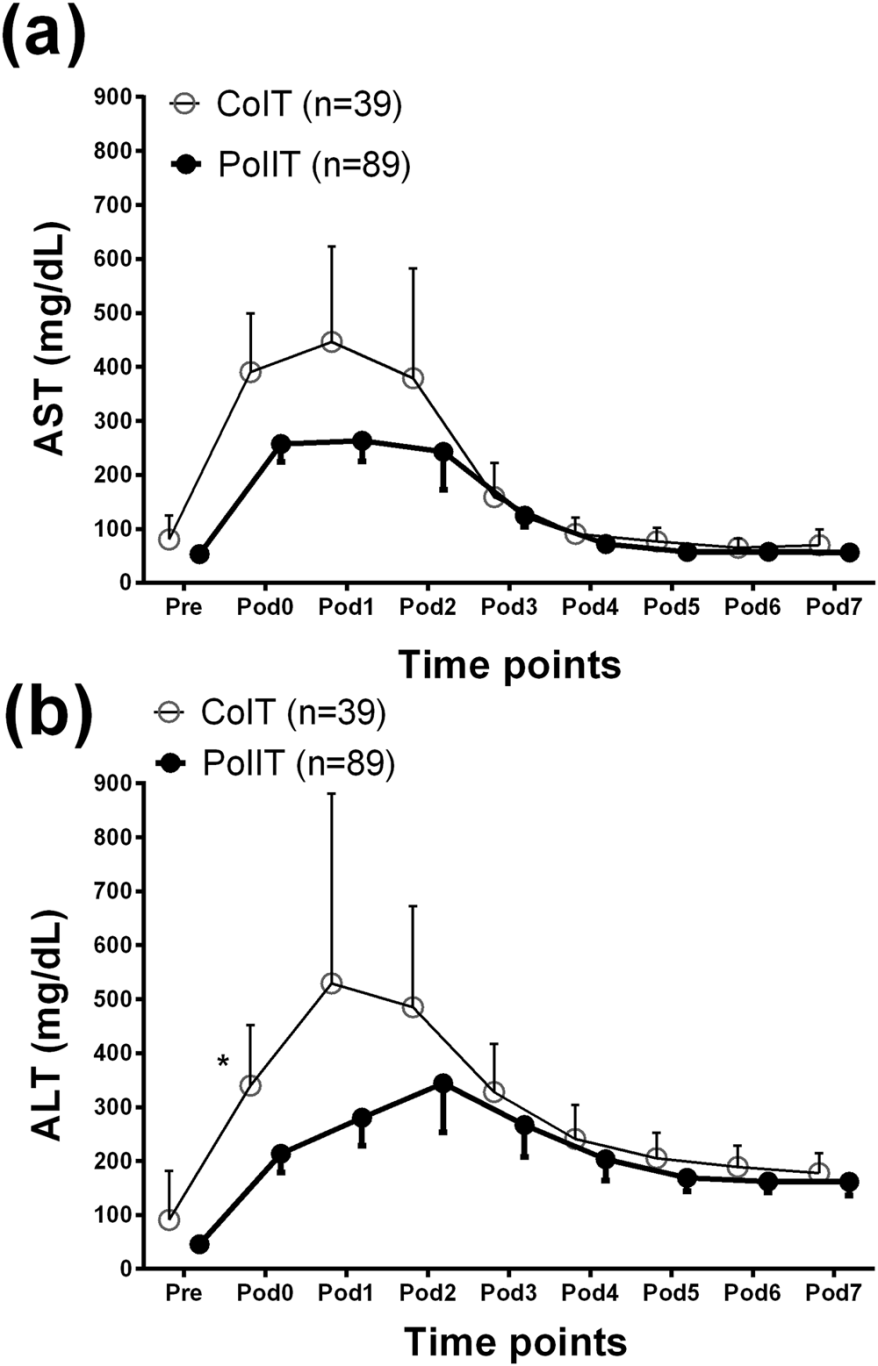
Perioperative changes in (**a**) aspartate transaminase (AST) and (**b**) alanine transaminase (ALT). Pre, the day before surgery; Pod, postoperative day. *p < 0.05 after Bonferroni correction in mixed linear model analysis.

As shown in Fig. 4a, repeatedly measured postoperative ASTs were significantly lower in PoIIT group than in CoIT group p = 0.015, indicating less hepatocyte injury in relation to the use of the PoIIT. In particular, AST level immediately after ICU arrival was 257 ± 159 IU/L in PoIIT group and 390 ± 333 IU/L in CoIT group. Repeatedly measured ALTs were insignificantly lower in PoIIT group (p = 0.412) (Fig. 4b). In particular, ALT level immediately after ICU arrival was 213 ± 167 IU/L in PoIIT group and 340 ± 347 IU/L in CoIT group. As shown in Table 3, results of univariable analysis indicated that the risks of major infection (4.5% vs. 17.9%, OR = 0.22 [0.06–0.79], p = 0.020), prolonged mechanical ventilation (6.7% vs. 20.5%, OR = 0.28 [0.09–0.87], p = 0.028), and biliary stricture (5.6% vs. 20.5%, OR = 0.23 [0.07–0.76], p = 0.016) were significantly lower in PoIIT group. After adjustments for age, sex, and diabetes mellitus, the PoIIT was significantly associated with decreases in major infections (OR = 0.23 [0.06–0.85], p = 0.028), prolonged mechanical ventilation (OR = 0.29 [0.09–0.89], p = 0.031), and biliary stricture (OR = 0.23 [0.07–0.78], p = 0.018). Detailed results of multivariable analysis for each outcome variable are described in Supplementary Table S2.

**Table 3:** Postoperative complications in univariable and multivariable analyses.

	Conventional (n = 39)	Portland (n = 89)	Univariable analysis		Multivariable analysis	
			OR	P	^†^OR	P
Major infection^*^	7 (17.9)	4 (4.5)	0.22 [0.06–0.79]	0.020	0.23 [0.06–0.85]	0.028
Surgical site infection	5 (12.8)	10 (11.2)	0.86 [0.27–2.71]	0.798	0.93 [0.29–3.01]	0.909
Cytomegalovirus infection	28 (71.8)	56 (62.9)	0.67 [0.29–1.51]	0.332	0.64 [0.28–1.46]	0.289
Gr IIIb-V complication	7 (17.9)	20 (22.5)	1.33 [0.51–3.45]	0.564	1.33 [0.51–3.47]	0.564
Bile leak	7 (17.9)	11(12.4)	0.65 [0.23–1.81]	0.405	0.64 [0.22–1.85]	0.406
Biliary stricture	8 (20.5)	5 (5.6)	0.23 [0.07–0.76]	0.016	0.23 [0.07–0.78]	0.018
Respiratory complication	11 (28.2)	20 (22.5)	0.74 [0.31–1.74]	0.487	0.75 [0.31–1.83]	0.521
Mechanical ventilation >24 hours	8 (20.5)	6 (6.7)	0.28 [0.09–0.87]	0.028	0.29 [0.09–0.89]	0.031
Acute kidney injury within 48 hours	9 (23.1)	20 (22.5)	0.97 [0.39–2.37]	0.940	0.95 [0.39–2.34]	0.915

Data are presented as frequency (%) and odds ratio (OR) with 95% confidence interval.

^*^Septicemia, peritonitis, pneumonia and tissue-invasive cytomegalovirus disease. ^†^Adjusted for age, sex, and diabetes mellitus.

## Discussion

In this study, we demonstrated that the PoIIT was superior to our CoIT for controlling acute hyperglycemic disturbance after graft reperfusion and for preventing intraoperative hyperglycemia during the postreperfusion phase in living donor liver transplantations. Hyperglycemia risk was reduced by 60% in relation to the use of the PoIIT (22.5% vs. 53.8%). Although greater amounts of insulin were used with the PoIIT, no recipients developed clinically relevant hypoglycemia. PoIIT showed an additional advantage by preventing increases in potassium concentration after graft reperfusion. Moreover, the use of the PoIIT was associated with improved post-transplant clinical courses in terms of hepatocyte injury, major infection, prolonged mechanical ventilation, and biliary stricture.

Among the various IITs^16^, our team selected the PoIIT because it was designed for surgical patients, while many IITs are for medical ICU patients, and because the PoIIT has been well-validated as an effective intraoperative glycemic protocol in cardiac surgeries during which glycemic environment and patient insulin sensitivity change considerably during surgery^13,14^. Moreover, a previous study has suggested that hypoglycemia risk is lower in the PoIIT than in other IITs, particularly when insulin sensitivity is impaired^16^. Nonetheless, it was unclear whether the PoIIT would be effective for use in liver transplantation. Some studies of patients undergoing liver surgeries have highlighted the necessity of developing better intraoperative glycemic strategies by reporting the difficulties in controlling BGC and negative clinical impacts of intraoperative hyperglycemia^4,5,30^. In a previous study comparing 60 liver transplant recipients with mean intraoperative BGC of <150 mg/dL and 124 recipients with mean intraoperative BGC of ≥150 mg/dL, higher intraoperative BGC was associated with post-transplant infections and mortality^5^. Another study of 680 recipients demonstrated that intraoperative hyperglycemia (BGC > 200 mg/dL) was associated with surgical site infection^4^. Despite the need for better intraoperative glycemic protocols for liver transplantation, there have been no studies evaluating the efficacy and/or safety of particular insulin infusion protocols.

The mechanisms underlying the association of the use of PoIIT with improved post-transplant clinical outcomes might be attributable to decreased intraoperative BGCs and hyperglycemia. First, acute transient hyperglycemia or BGC fluctuation even during short periods disturbs the innate immune system by inhibiting neutrophil migration, phagocytosis, and complement function, stimulating inflammatory cytokines, and decreasing microvascular reactivity^7,8^. The deterioration of innate immune response may promote infection progress. Second, previous research demonstrated that only a transient period of acute hyperglycemia is necessary to aggravate ischemia reperfusion injury via oxidative stress and impaired tissue microcirculation^3,9^. A previous study of critically ill patients demonstrated that hepatocyte mitochondrial ultrastructure and function can be protected by preventing acute hyperglycemia with tight glycemic control^31^. Moreover, insulin may directly mitigate hepatocyte injury by serving as a scavenger of free radicals generated during the ischemia reperfusion process^32^. Third, decreased hepatic ischemia reperfusion injury could also benefit the bile duct^23^. Ischemia reperfusion injury to the microvasculature of the bile duct arteriolar plexus causes biliary epithelial cell damage; consequently, inflammatory cells penetrate between epithelial cells and basement membranes, and result in biliary stricture^33^, Moreover, insulin is an essential molecule for the regeneration of hepatic tissues including the bile duct^34,35^. A previous study in rats demonstrated that the degree of liver regeneration after hepatectomy was lower in subjects with decreased insulin sensitivity and suggested the benefit of insulin administration^36^. Another study demonstrated that early graft regeneration after LDLT was improved in relation to postoperative insulin administration^37^. Fourth, acute hyperglycemia-induced oxidative stress is systemic and can affect various tissues and organs in remote areas including the diaphragm^38^, which supports the association between the use of the PoIIT and decreased risk of prolonged mechanical ventilation. In addition, a direct anabolic effect of insulin on respiratory muscles may play a role: previous research demonstrated that insulin administration promotes skeletal muscle protein uptake and synthesis, improving skeletal muscle function^39^.

This study had several limitations. First, due to its retrospective nature, we are unable to establish a direct cause-and-effect relationship between the use of the PoIIT and improved post-transplant outcomes, although some of the underlying mechanisms can be hypothesized based on previous studies. In addition, we were unable to include more variables in the multivariable model due to the limited event number^40^. Thus, the association between strict glycemic management and post-transplant outcomes warrants further research with sufficient sample size. Nonetheless, we performed the Bonferroni correction and the Benjamini-Hochberg procedure to decrease the risk of type I error. Moreover, the sufficient sample size to assess the primary outcome was an advantage of the current study: the power of the 50% reduction of an assumed intraoperative hyperglycemia rate with the PoIIT was 83%. Second, there was a potential risk of selection bias because the use of the PoIIT or CoIT was determined at the discretion of attending anesthetists. In this regard, we tested and confirmed the absence of significant differences among 4 attending anesthetists who covered all recipients evaluated in the current study regarding graft quantity and quality, donor factors, recipient factors, and preoperative laboratory findings (Supplementary Table S3). Third, only 25.8% of recipients with the PoIIT reached the target range (80–120 mg/dL) before ICU arrival, during about 4 hours after the initiation of the PoIIT, most likely due to profound hepatic injury and severe insulin insensitivity. Thus, it remains unclear that how fast the target BGC range can be achieved and how safely the target range can be maintained once it is achieved. It should be noted that IIT-induced severe hypoglycemia and hypokalemia can cause poor outcomes^17,41^. In addition, identifying the optimal time window for the use of the PoIIT warrants further research. Nonetheless, the results of the current study suggest that transient use of PoIIT during the intraoperative postreperfusion phase and prevention of acute hyperglycemia for the critical time window may provide clinical benefits.

The use of the PoIIT during the intraoperative postreperfusion phase was associated with decreases in intraoperative hyperglycemia and postoperative complications such as infections, prolonged mechanical ventilation, and biliary stricture. We observed no increases in the risks of hypoglycemia or hypokalemia in relation to the use of the PoIIT. Our findings suggest that the PoIIT effectively and safely controls acute hyperglycemic change after graft reperfusion and prevents hyperglycemia during LDLT, resulting in potential clinical benefits.

## Electronic supplementary material

Supplementary tables and figures

## Data Availability

The datasets generated during and/or analyzed during the current study are available from the corresponding author on reasonable request.
