# Supplementary material for: Portland Intensive Insulin Therapy During Living Donor Liver Transplantation: Association with Postreperfusion Hyperglycemia and Clinical Outcomes
Source: Sci Rep. 2018 Nov 2;8:16306. doi: 10.1038/s41598-018-34655-6 (PMC6214899; doi:10.1038/s41598-018-34655-6)

**Portland Intensive Insulin Therapy During Living Donor Liver Transplantation: Association with Postreperfusion Hyperglycemia and Clinical Outcomes**

RyungA Kang,^1^ Sangbin Han,^1*^ Kyo Won Lee,^2^ Gaab Soo Kim,^1^ Soo Joo Choi,^1^ Justin S. Ko,^1^

Sang Hyun Lee,^1^ Mi Sook Gwak^1^

^1^Department of Anesthesiology and Pain Medicine, Samsung Medical Center, Sungkyunkwan University School of Medicine, Seoul, Korea

^2^Department of Surgery, Samsung Medical Center, Sungkyunkwan University School of Medicine, Seoul, Korea

**Table of Contents** for Supplementary Materials

| Supplementary Table S1 | 2 |
| --- | --- |
| Supplementary Table S2 | 3-4 |
| Supplementary Table S3 | 5-6 |
| Supplementary Figure S1 | 7-9 |
| Supplementary Figure S2 | 10 |

**SUPPLEMENTARY TABLE S1.** The proportion of the Portland intensive insulin therapy protocol, instead of conventional protocol, varied by anesthetists and the period of operation.

|  | Anesthetists | | | | *P* |
| --- | --- | --- | --- | --- | --- |
|  | GSK (n=44) | MSK (n=42) | JK (n=22) | SH (n=20) |  |
| The use of the Portland protocol | 33 (75.0) | 18 (42.9) | 21 (95.5) | 17 (85.0) | 0.001 |
| Jan. 2015–Dec. 2015 | 12/22 (54.5) | 5/18 (27.8) | 4/5 (80) | 12/14 (85.7) |  |
| Jan. 2016–Dec. 2016 | 16/17 (94.1) | 5/15 (33.3) | 8/8 (100) | 4/5 (80) |  |
| Jan. 2017–Jul. 2017 | 5/5 (100) | 8/9 (88.9) | 9/9 (100) | 1/1 (100) |  |

Data are presented as frequency (%).

**SUPPLEMENTARY TABLE S2.** Detailed results of multivariable analysis for each outcome variable.

| Outcomes |  | Multivariable analysis | |
| --- | --- | --- | --- |
|  | Variables | OR | P |
| Major infection^*^ | PoIIT (vs. CoIT) | 0.23 [0.06-0.85] | 0.028 |
|  | Age | 0.98 [0.91-1.06] | 0.612 |
|  | Male sex | 1.05 [0.25-4.52] | 0.944 |
|  | Diabetes | 2.98 [0.80-11.13] | 0.104 |
| Surgical site infection | PoIIT (vs. CoIT) | 0.93 [0.28-3.07] | 0.908 |
|  | Age | 0.95 [0.89-1.01] | 0.123 |
|  | Male sex | 1.76 [0.44-6.97] | 0.423 |
|  | Diabetes | 2.96 [0.91-9.59] | 0.071 |
| Cytomegalovirus infection | PoIIT (vs. CoIT) | 0.64 [0.28-1.46] | 0.289 |
|  | Age | 1.00 [0.95-1.05] | 0.980 |
|  | Male sex | 1.19 [0.53-2.70] | 0.676 |
|  | Diabetes | 0.65 [0.27-1.54] | 0.324 |
| Gr IIIb-V complication | PoIIT (vs. CoIT) | 1.33 [0.51-3.47] | 0.567 |
|  | Age | 1.00 [0.94-1.05] | 0.918 |
|  | Male sex | 0.87 [0.34-2.21] | 0.762 |
|  | Diabetes | 1.00 [0.36-2.80] | >0.999 |
| Bile leak | PoIIT (vs. CoIT) | 0.66 [0.23-1.91] | 0.441 |
|  | Age | 1.03 [0.96-1.12] | 0.428 |
|  | Male sex | 7.74 [0.98-61.00] | 0.052 |
|  | Diabetes | 1.30 [0.41-4.17] | 0.656 |
| Biliary stricture | PoIIT (vs. CoIT) | 0.23 [0.07-0.77] | 0.018 |
|  | Age | 0.99 [0.91-1.08] | 0.852 |
|  | Male sex | 5.66 [0.68-47.19] | 0.109 |
|  | Diabetes | 2.19 [0.61-7.84] | 0.227 |
| Respiratory complication | PoIIT (vs. CoIT) | 0.75 [0.31-1.85] | 0.534 |
|  | Age | 1.03 [0.97-1.09] | 0.332 |
|  | Male sex | 0.27 [0.11-0.64] | 0.003 |
|  | Diabetes | 1.19 [0.44-3.19] | 0.732 |
| Mechanical ventilation >24 hours | PoIIT (vs. CoIT) | 0.28 [0.09-0.89] | 0.030 |
|  | Age | 0.98 [0.91-1.05] | 0.496 |
|  | Male sex | 0.66 [0.20-2.23] | 0.507 |
|  | Diabetes | 1.30 [0.36-4.70] | 0.693 |
| Acute kidney injury within 48 hours | PoIIT (vs. CoIT) | 0.94 [0.38-2.31] | 0.884 |
|  | Age | 0.98 [0.93-1.03] | 0.421 |
|  | Male sex | 1.27 [0.48-3.33] | 0.628 |
|  | Diabetes | 0.89 [0.32-2.48] | 0.826 |

Data are presented as frequency (%) and odds ratio (OR) with 95% confidence interval.

^*^Septicemia, peritonitis, pneumonia and tissue-invasive cytomegalovirus disease. CoIT, conventional insulin therapy; PoIIT, Portland intensive insulin therapy.

**SUPPLEMENTARY TABLE S3.** Clinical characteristics of recipients according to the attending anesthetists.

|  | GSK (n=44) | MSK (n=42) | JK (n=22) | SH (n=20) | *P* |
| --- | --- | --- | --- | --- | --- |
| **Graft factors** |  |  |  |  |  |
| Age (years) | 33±12 | 34±12 | 30±14 | 32±11 | 0.755 |
| Male sex | 21 (47.7) | 26 (61.9) | 16 (72.7) | 10 (50) | 0.212 |
| BMI(kg/m^2^) | 23±3 | 24±3 | 24±2 | 24±3 | 0.306 |
| Macrosteatosis>5% | 16 (36.4) | 16 (38.1) | 8 (36.4) | 5 (25.0) | 0.791 |
| **Recipient factors** |  |  |  |  |  |
| Age (years) | 55±7 | 56±9 | 56±6 | 56±8 | 0.950 |
| Male sex | 31 (70.5) | 30 (71.4) | 19 (86.4) | 13 (65.0) | 0.433 |
| Body mass index (kg/m^2^) | 25±5 | 24±3 | 25±4 | 24±4 | 0.957 |
| Hypertension | 6 (13.6) | 2 (4.8) | 4 (18.2) | 1 (5.0) | 0.257 |
| Diabetes | 13 (29.5) | 8 (19.0) | 3 (13.6) | 5 (25.0) | 0.773 |
| Primary etiology  (Hepatitis B/ Hepatitis C/ Alcohol/others) | 27 (61.4)/ 6 (13.6)/ 1 (2.3)/ 10 (22.7) | 27 (64.3)/ 2 (4.8)/ 2 (4.8)/ 11 (26.2) | 16 (72.7)/ 4 (18.2)/ 1 (4.5)/ 1 (4.5) | 15 (75)/ 2 (10)/ 1 (5)/ 2 (10) | 0.481 |
| Hepatocellular carcinoma | 28 (63.6) | 27 (64.3) | 19 (86.4) | 14 (70.0) | 0.259 |
| Graft-to-recipient ratio | 1.0±0.3 | 1.1±0.3 | 1.1±0.3 | 1.1±0.3 | 0.644 |
| MELD score | 14±7 | 15±9 | 16±9 | 15±9 | 0.865 |
| Cold ischemia time (minutes) | 90±22 | 91±22 | 96±25 | 98±20 | 0.545 |
| Preoperative laboratory findings |  |  |  |  |  |
| Glucose (mg/dL) | 115±49 | 110±36 | 101±21 | 109±31 | 0.550 |
| Potassium (mEq/L) | 4.1±0.4 | 3.9±0.4 | 4.1±0.4 | 4.1±0.4 | 0.148 |
| Albumin(mg/dL) | 3.4±0.6 | 3.5±0.6 | 3.2±0.5 | 3.4±0.7 | 0.472 |
| Creatinine (mg/dL) | 0.9±0.3 | 0.8±0.3 | 0.9±0.3 | 0.9±0.5 | 0.606 |
| Sodium (mmol/L) | 138±6 | 138±5 | 138±5 | 140±6 | 0.809 |
| High sensitivity CRP (mg/dL) | 0.7±1.3 | 0.7±1.3 | 0.7±1.6 | 0.4±0.5 | 0.777 |
| Neutrophil-to-lymphocyte ratio | 3.1±3.0 | 3.3±5.3 | 3.0±1.9 | 2.7±2.3 | 0.939 |
| Total bilirubin (mg/dL) | 4.7±8.1 | 6.3±11.3 | 6.8±12.8 | 4.4±10.2 | 0.775 |
| Lactate | 1.9±2.1 | 1.5±0.8 | 1.6±0.6 | 1.9±1.1 | 0.866 |
| Aspartate transaminase (U/L) | 51±51 | 65±112 | 62±49 | 75±106 | 0.747 |
| Alanine transaminase (U/L) | 38±36 | 52±117 | 63±102 | 116±356 | 0.349 |

Data are presented as mean±standard deviations or number (%). CRP, C-reactive protein; MELD, model for end stage liver disease.

**SUPPLEMENTARY FIGURE S1.** Portland protocol during the reperfusion phase in liver transplantation (version 2008.2; ICU phase 3; target blood glucose 80-120 mg/dL)

**▣ Preparation an Insulin Infusion ▣**

1. Mix 1 unit regular insulin per 1 ml 0.9% normal saline. Administer via infusion pump.
2. Target blood glucose concentrations: 80-120 mg/dL

**▣ Blood Glucose Concentration (BGC) Monitoring ▣**

1. Check BGC predetermined time points
2. Start “The Portland protocol” for **5 minutes after reperfusion BGC > 125 mg/dL**, including “non-diabetes” patients
3. If any of the following occur, notify the attending staff, consider the target BGC change
4. BGC <70 mg/dL
5. K+ concentration <3.0 mEq/L

**▣ Insulin Initiation Dose ▣**

| Blood glucose | IV Regular Insulin Syringe Bolus | Initial Regular Insulin Rate:  Units/Hour | |
| --- | --- | --- | --- |
|  | | NIDDM or non-DM | IDDM |
| 110 to 124 mg/dL | 0 Units | None | None |
| 125 to 150 mg/dL | 2 → **For DM patients only*** | 1 Unit / Hour | 2 Units / Hour |
| 151 to 180 mg/dL | 4 Units | 2 Units / Hour | 3.5 Units / Hour |
| 181 to 240 mg/dL | 6 Units | 3.5 Units / Hour | 5 Units / Hour |
| 241 to 300 mg/dL | 8 Units | 5 Units / Hour | 6.5 Units / Hour |
| 301 to 360 mg/dL | 12 Units | 6.5 Units / Hour | 8 Units / Hour |
| Greater than 360 mg/dL | 16 Units | 8 Units / Hour | 10 Units / Hour |

**▣ Changing the Insulin Infusion Rate ▣**

| **Blood**  **Glucose (BG)** | **Note: If ANY BG is less than 40mg/dl or greater than 450mg/dl, obtain confirmatory laboratory BG**  **Action:** |
| --- | --- |
| **<50 mg/dL** | **Stop Insulin**  Give 15 ml of D50W IV; If <40 give 25 ml of D50W IV  Recheck BG every 30 minutes until greater than 80mg/dl  If next BG is <50mg/dl: Double amount of previous treatment; If next BG is 50 – 65 mg/dl repeat treatment  When BG greater than 90mg/dl: Restart Insulin rate at 50% of previous rate & recheck BG in 30 minutes |
| **50-64 mg/dL** | **Stop Insulin**  If Previous BG greater than 100 mg/dl OR if symptomatic from hypoglycemia: give 15 ml of D50W IV  Recheck BG every 30 minutes until greater than 80mg/dl  If next BG remains 50-64 mg/dl : Repeat previous treatment  When BG greater than 90mg/dl: Restart Insulin rate at 50% of previous rate & recheck BG in 30 minutes |
| **65-79 mg/dL** | If greater than last test: Decrease rate by 0.2units / Hour  If lower than last BG by more than 30 mg/dl: Stop drip & recheck BG in 30 minutes (see bold * order)  If lower than last BG by 15 – 30 mg/dl: Decrease rate by HALF (50%) & recheck BG in 30 minutes  If lower than last BG by 7 – 14 mg/dl: Decrease rate by 0.5 Units / Hour  If equal to last BG or lower than last BG by less than 7 mg/dl: Decrease rate by 0.3 Units / Hour  ***If infusion turned off, recheck BG in 30 min, when BG greater than 90mg/dl restart at 50% of previous rate &recheck BG in 30 minutes**  Recheck BG every 30 minutes until greater than 80mg/dl |
| **80-120 mg/dL**  **(Target range)** | If higher than last BG by more than 10mg/dl: Increase rate by 0.5 Units / Hour  If lower than last BG by more than 40 mg/dl: **Stop drip** & recheck BG in 30 minutes (see bold ** order)  If lower than last BG by 21–40 mg/dl: Decrease rate by HALF (50%) & recheck BG in 30 minutes  If lower than last BG by 10-20mg/dl: Decrease rate by 0.5 Units / Hour  **** If infusion turned off, recheck BG 30 min, if /when BG greater than 120mg/dl restart at 50% of previous rate**  If within 10mg/dl of last BG same rate… unless the following applies:  **FOR ANY BG** in this range (even if within 10 mg/dl of last test) the following **ALWAYS** applies:  º BG has consistently decreased each of last 4 measurements: Decrease rate by an **additional** 0.3 Units / Hour  º BG has consistently increased each of last 4 measurements: Increase rate by an **additional** 0.2 Units / Hour |
| **121-135 mg/dL** | If higher than last BG by more than 50mg/dl: Increase rate by 2 Units/Hour  If higher than last BG by 20 - 50mg/dl: Increase rate by 1 Unit / Hour  If higher than last BG by 0 - 20mg/dl: Increase rate by 0.5 Units / Hour  If lower than last BG by 1 - 20 mg/dl: Same rate  If lower than last BG by 21 - 40mg/dl: Decrease rate by 1 Unit / Hour  If lower than last BG by 41 – 60 mg/dl: Decrease rate by HALF (50%) and recheck BG in 30 minutes  If lower than last BG by more than 60 mg/dl: **Stop drip & recheck BG in 30 minutes** (see bold ** order below)  ****If infusion turned off, recheck BG 30 min, if /when BG greater than 125mg/dl restart at 50% of previous rate** |
| **136-150 mg/dL** | If higher than last BG by more than 30mg/dl: Increase rate by 2 Units/Hour & bolus with 3 units IV  If higher than last BG by 0 - 30mg/dl: Increase rate by 1 Unit / Hour & bolus with 2 units IV  If lower than last BG by 1 – 20: Increase rate by 1 Unit / Hour & bolus with 2 units IV  If lower than last BG by 21 to 50mg/dl: Same rate  If lower than last BG by 51 – 80mg/dl: Decrease rate by HALF (50%) and recheck BG in 30 minutes  If lower than last BG by more than 80mg/dl: Stop drip & recheck BG in 30 minutes (see bold ** order below)  ****If infusion turned off, recheck BG 30 min, if /when BG greater than 125mg/dl restart at 50% of previous rate** |
| **181-240 mg/dL** | If lower than last BG by more than 100 mg/dl: Decrease rate by HALF (50%)  If lower than last BG by 50 – 100 mg/dl: Continue same rate  If lower than last BG by less than 50mg/dl OR higher than last BG:  ºBOLUS with 6 units Regular Insulin IV AND Increase Insulin rate by 2 Units / Hour  If BG remains 181 - 240 mg/dl and has not decreased after 3 consecutive increases in Insulin:  a. Give DOUBLE previous IV BOLUS dose up to a maximum of 24 units **AND**  b. DOUBLE Insulin drip rate -- up to a maximum of 20 units / hour  c. If on 20 units/hour and no response after 4 maximum boluses – CALL MD for further orders  Recheck BG in 30 minutes. Repeat BG every 30 minutes until less than 150mg/dl |
| **>240 mg/dL** | If lower than last BG by more than 150 mg/dl: Decrease rate by HALF (50%)  If lower than last BG by 101-150mg/dl : Same rate  If lower than last BG by 0- 100mg/dl OR if higher than last BG:  º BOLUS with 10 Units Regular Insulin IV AND DOUBLE Insulin rate up to a maximum of 30 units / hour  If BG remains greater than 240 mg/dl and has not decreased after 3 consecutive increases in Insulin:  d. Give DOUBLE previous IV BOLUS dose up to a maximum of 40 units AND  e. DOUBLE Insulin drip rate -- up to a maximum of 30 units / hour  f. If on 30 units/hour and no response after 4 maximum boluses – CALL MD for further orders  Recheck BG in 30 minutes. Repeat BG every 30 minutes until less than 150mg/dl |

| **Time points** | **Glucose (mg/dL)** | **Potassium (mEq/L)** | **RI bolus (IU)** | **RI continuous (IU/hr)** |
| --- | --- | --- | --- | --- |
| 10 min before reperfusion |  |  | X | X |
| 5 min after reperfusion |  |  |  |  |
| 30 min after reperfusion |  |  |  |  |
| 1 hour after reperfusion |  |  |  |  |
| 2 hours after reperfusion |  |  |  |  |
| 3 hours after reperfusion |  |  |  |  |
| 4 hours after reperfusion |  |  |  |  |
| ICU arrival |  |  | X | X |

**▣ Flow Sheet ▣**

**SUPPLEMENTARY FIGURE S2.** Comparable postoperative (a) blood glucose concentrations and (b) blood potassium concentrations between recipients treated with the conventional insulin therapy (CoIT) and recipients treated with the Portland intensive insulin therapy (PoIIT).


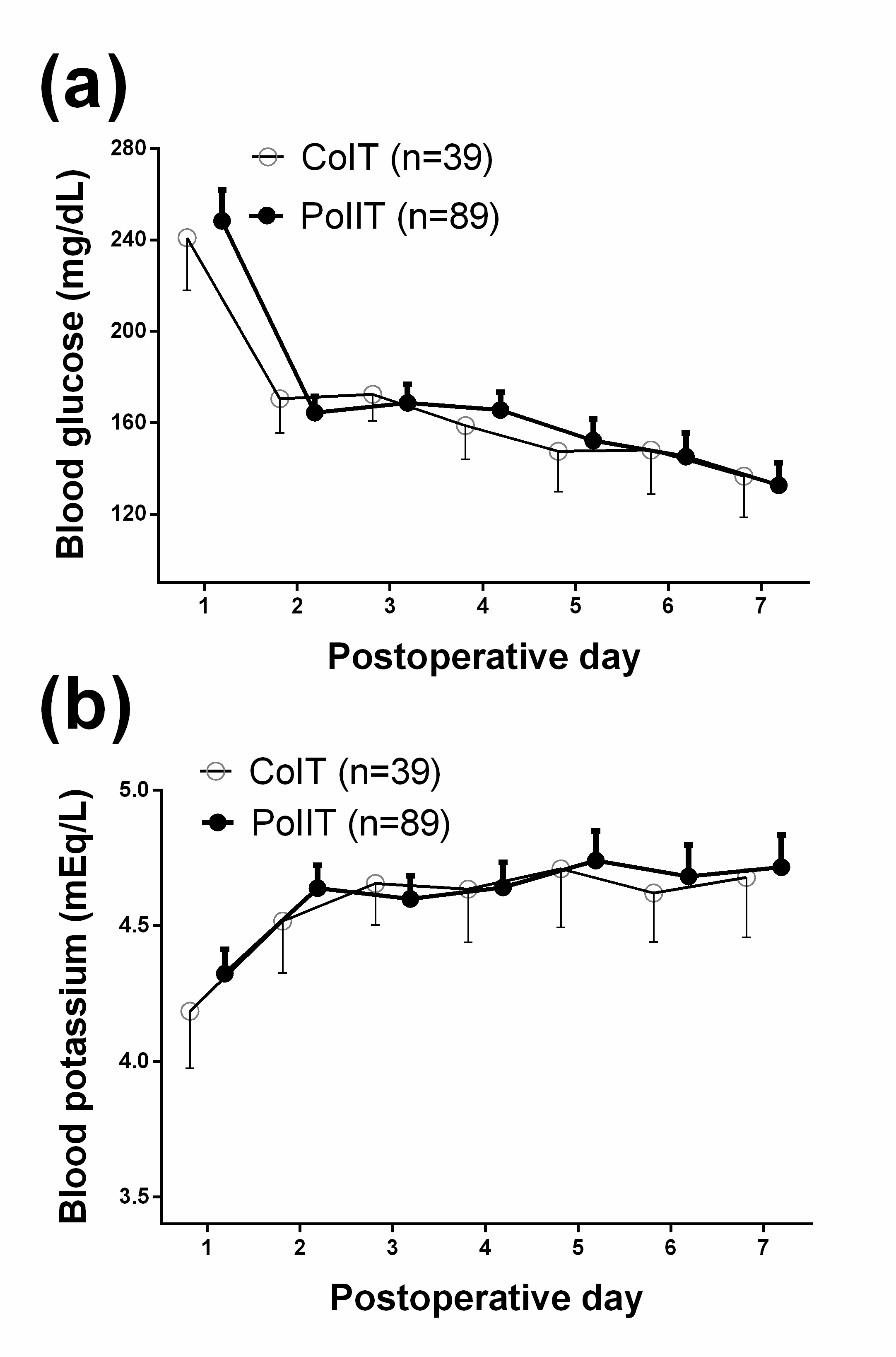

Supplement: Supplementary file 1 — Supplementary tables and figures [file 41598_2018_34655_MOESM1_ESM.doc]
